# De Novo Myasthenia Gravis Induced by Atezolizumab in a Patient with Urothelial Carcinoma

**DOI:** 10.7759/cureus.5002

**Published:** 2019-06-25

**Authors:** Smathorn Thakolwiboon, Amputch Karukote, Henrik Wilms

**Affiliations:** 1 Neurology, Texas Tech University Health Sciences Center, Lubbock, USA

**Keywords:** myasthenia gravis, immune checkpoint inhibitor, immune-related adverse events, atezolizumab, program cell death ligand-1, pd-l1 inhibitor

## Abstract

The programmed cell death ligand-1 antibody, atezolizumab, is an immune checkpoint inhibitor approved for the treatment of various cancers. Herein, we describe a case of an 87-year-old man with advanced urothelial carcinoma. After surgery, atezolizumab was given. Subsequently, he developed generalized myasthenia gravis (MG) with elevated creatinine kinase and positive anti-striated muscle antibody. Although intravenous immunoglobulin was started, the patient developed cardiac arrhythmia and arrest. Our case not only reported MG as an immune-related adverse event of atezolizumab but also emphasized the significance of the programmed cell death-1 pathway in the pathogenesis of MG.

## Introduction

Immune checkpoint inhibition is a novel therapeutic strategy for several advanced cancers. Immune checkpoint inhibitors (ICIs) block the molecules which inactivate T-cells and enhance an immune response to tumor cells. On the other hand, by unleashing T cells ICIs can trigger immune-related adverse events (IRAEs) [1].

Atezolizumab is a fully humanized immunoglobulin monoclonal antibody that blocks the interaction of the programmed cell death ligand-1 (PD-L1) with the programmed cell death receptor-1 (PD-1) and CD80, which is one of the immune escape mechanisms of tumor cells. Atezolizumab has been approved for the treatment of advanced non-small cell lung cancer (NSCLC), urothelial carcinoma, triple-negative breast cancer and small cell lung cancer (SCLC) [2]. Recently, various autoimmune conditions have been described with atezolizumab including colitis, hepatitis, pneumonitis, hypophysitis as well as autoimmune encephalitis [3-4].

Myasthenia gravis (MG) is an autoimmune disorder of neuromuscular junctions and usually manifests with fatigable weakness. It was reported in association with several ICIs including pembrolizumab, nivolumab, and ipilimumab [5]. Herein, we described a case of new-onset MG in a patient treated with atezolizumab.

## Case presentation

An 87-year-old Caucasian man with hypertension, dyslipidemia, and chronic kidney disease had recently been diagnosed with muscle-invasive urothelial carcinoma of the bladder. He underwent radical cystoprostatectomy and bilateral lymph node dissection. Subsequently, intravenous atezolizumab 1200 mg was given every three weeks.

After the second dose, he started having double vision and ptosis, followed by proximal muscle weakness and nasal voice which were worse with prolonged use. Despite being treated with prednisone 60 mg daily for seven days, the symptoms continued to progress. Therefore, he was transferred to our facility.

At presentation, vital signs were normal. Oxygen saturation at room air was 93%. He was alert and fully oriented. The cardiovascular and respiratory examinations were normal. Neurological examination showed severe external ophthalmoplegia and bilateral incomplete ptosis, which was improved after the placement of an instant cold pack. Neck flexion and extension powers were grade 4/5 and 5/5, respectively. The motor power in the four extremities was grade 3/5 for proximal and 4/5 for distal muscles. Deep tendon reflexes were normal. The sensory function was intact. Myasthenia gravis composite score was 17.

**Figure 1:**
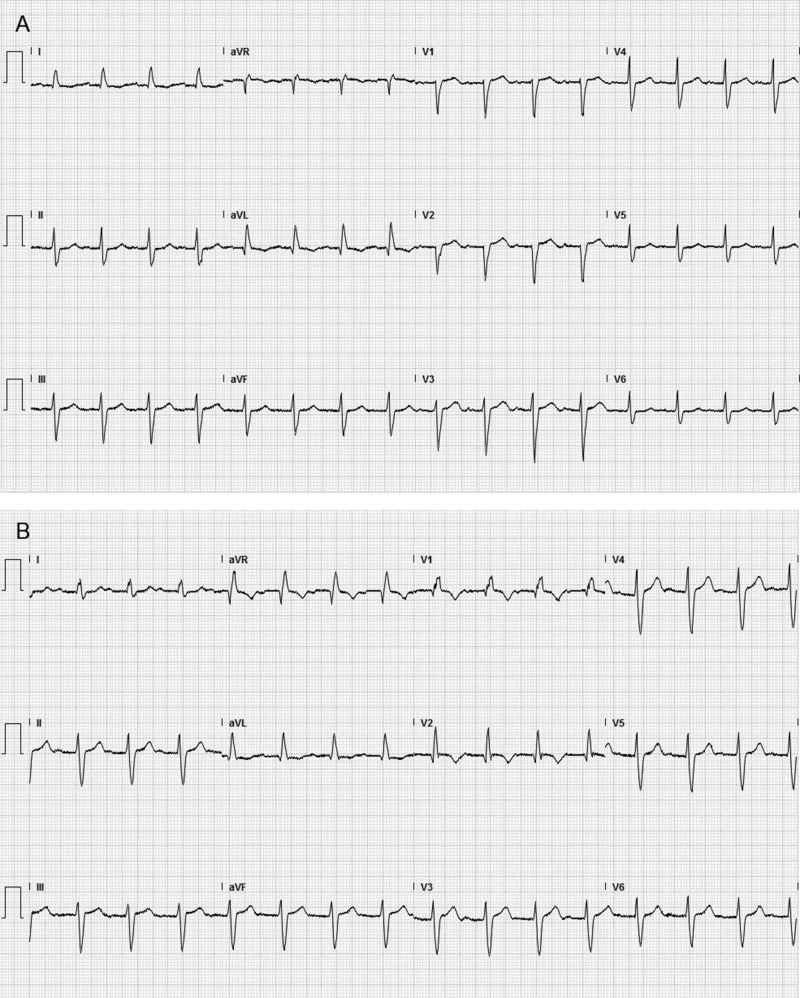
A: Electrocardiogram before treatment with atezolizumab showed only first-degree atrioventricular block. B: New right bundle branch block and left anterior fascicular block were seen after atezolizumab treatment

Laboratory studies showed elevated creatinine kinase (CK) at 1,542 U/L. C-reactive protein was 0.4 mg/dl. Antinuclear antibody, rheumatoid factor, cyclic citrulline peptide as well as, SS-A, SS-B, proteinase-3, and myeloperoxidase antibodies were negative. Electrocardiogram (ECG) showed a new right bundle branch block (RBBB) and left anterior fascicular block (Figure 1). Computed tomography of the chest showed no thymoma. Magnetic resonance imaging of the brain showed no stroke or brain metastasis.

Based on history, neurological syndrome on examination, lab findings, history of drug exposure, and exclusion of other confounding factors, the patient was diagnosed with new-onset Atezolizumab-induced class III MG based on the classification by the Myasthenia Gravis Foundation of America. Intravenous immunoglobulin (IVIG) 0.4 g/kg daily was started and planned to be given for five days. Low-dose oral pyridostigmine was later started. Muscle strength was improved after the administration of pyridostigmine as well.

Unfortunately, the patient developed cardiac arrest on the third day of admission. Complying with the patient's advanced directive, the family declined cardiopulmonary resuscitation. Prior to cardiac arrest, the patient had no sign of respiratory distress or chest pain. ECG monitoring showed occasional premature ventricular complexes and then asystole.

Later, the myasthenia and myositis antibodies panels were reported positive for an anti-striated muscle antibody at 1:40. Acetylcholine receptor (AChR)-blocking antibody was 21% inhibition. AChR binding and modulating antibodies were undetectable. muscle-specific tyrosine kinase and voltage-gated calcium channel antibodies were negative. All myositis antibodies were undetectable.

## Discussion

Immune checkpoints consist of various inhibitory pathways for the regulation of the immune system and the prevention of autoimmune disease. In many types of cancer, tumor cells hijack this inhibitory mechanism to escape the immune system. In current clinical practice, there are three classes of ICIs including Cytotoxic T lymphocyte-associated protein-4 (CTLA-4), PD-1, and PD-L1 inhibitors. These novel molecules have been approved for several cancers such as melanoma, NSCLC, renal cell carcinoma as well as urothelial cancer [6].

In the physiological state, CTLA-4 and PD-1 axes play a major role in the inhibition of autoreactive T cells. Polymorphisms of CTLA-4 and PD-1 are associated with various autoimmune diseases [1]. In the tumor environment, these molecules are overexpressed and inhibit the immune response as well [7]. The blockade of these proteins reactivates cytotoxic T cells to destroy tumor cells. However, unbalancing the immune system by blocking immune checkpoints favors the development of autoimmune disorders.

CTLA-4 and PD-1 inhibitors, as well as their combinations, have been reported to trigger both new onset and exacerbation of MG with a significantly high mortality rate [5]. As compared to cases of idiopathic MG, concomitant myositis and myocarditis, as well as mortality, were significantly higher in ICI-induced MG [8].

Atezolizumab was previously reported with exacerbation of MG which complicated with hypercapnic respiratory failure and required intubation [9]. Our case is the first reported case of atezolizumab-related new onset MG. Elevation of serum CK, as seen in our case, is frequent in ICI-associated MG and may indicate concomitant myositis [5]. New RBBB with left anterior fascicular block and progressive cardiac arrhythmia prior to cardiac arrest raised the possibility of myocardium involvement. However, confirmation of muscle and myocardium involvement by autopsy was not performed according to the family’s wish. 

In MG, the prior study showed significant overexpression of PD-1 on CD4+ T cells and PD-L1 on CD14+monocytes [10]. Moreover, overexpression of PD-L1 on muscle cells of MG patients was reported with a unimodal relationship with severity, measured by Quantitative myasthenia gravis score [11]. This case report demonstrated an MG causing by the blockage of the interaction between PD-1 and PD-L1 in human. These findings highlighted the regulatory role of the PD-1/PD-L1 pathway in the pathogenesis of MG. However, more studies are needed to understand the role of the PD-1/PD-L1 pathway in MG.

## Conclusions

ICIs are the novel therapeutic agents for cancers. On the other hand, these agents can trigger IRAEs. Our case highlights de novo MG as a severe IRAE of atezolizumab. As in the presented case, the previous case reports suggested that concomitant myositis and myocarditis are common in ICI-induced MG. Moreover, MG induced by ICIs is usually severe and associates with high mortality. Furthermore, our case provides further evidence for the functional significance of the PD-1/PD-L1 pathway in the pathogenesis of MG.
